# Mitochondrial transplantation therapy for ischemia reperfusion injury: a systematic review of animal and human studies

**DOI:** 10.1186/s12967-021-02878-3

**Published:** 2021-05-17

**Authors:** Kei Hayashida, Ryosuke Takegawa, Muhammad Shoaib, Tomoaki Aoki, Rishabh C. Choudhary, Cyrus E. Kuschner, Mitsuaki Nishikimi, Santiago J. Miyara, Daniel M. Rolston, Sara Guevara, Junhwan Kim, Koichiro Shinozaki, Ernesto P. Molmenti, Lance B. Becker

**Affiliations:** 1 The Feinstein Institutes for Medical Research, Northwell Health System, 350 Community Drive, Manhasset, NY USA; 2 Department of Emergency Medicine, North Shore University Hospital, Northwell Health System, 350 Community Dr, Manhasset, NY 11030 USA; 3 Zucker School of Medicine At Hofstra/Northwell, New York, NY USA; 4 Department of Surgery, Northwell Health, Manhasset, NY USA

**Keywords:** Systematic review, Mitochondria, Transplantation, Ischemia reperfusion injury, Translation science

## Abstract

**Background:**

Mitochondria are essential organelles that provide energy for cellular functions, participate in cellular signaling and growth, and facilitate cell death. Based on their multifactorial roles, mitochondria are also critical in the progression of critical illnesses. Transplantation of mitochondria has been reported as a potential promising approach to treat critical illnesses, particularly ischemia reperfusion injury (IRI). However, a systematic review of the relevant literature has not been conducted to date. Here, we systematically reviewed the animal and human studies relevant to IRI to summarize the evidence for mitochondrial transplantation.

**Methods:**

We searched MEDLINE, the Cochrane library, and Embase and performed a systematic review of mitochondrial transplantation for IRI in both preclinical and clinical studies. We developed a search strategy using a combination of keywords and Medical Subject Heading/Emtree terms. Studies including cell-mediated transfer of mitochondria as a transfer method were excluded. Data were extracted to a tailored template, and data synthesis was descriptive because the data were not suitable for meta-analysis.

**Results:**

Overall, we identified 20 animal studies and two human studies. Among animal studies, 14 (70%) studies focused on either brain or heart IRI. Both autograft and allograft mitochondrial transplantation were used in 17 (85%) animal studies. The designs of the animal studies were heterogeneous in terms of the route of administration, timing of transplantation, and dosage used. Twelve (60%) studies were performed in a blinded manner. All animal studies reported that mitochondrial transplantation markedly mitigated IRI in the target tissues, but there was variation in biological biomarkers and pathological changes. The human studies were conducted with a single-arm, unblinded design, in which autologous mitochondrial transplantation was applied to pediatric patients who required extracorporeal membrane oxygenation (ECMO) for IRI–associated myocardial dysfunction after cardiac surgery.

**Conclusion:**

The evidence gathered from our systematic review supports the potential beneficial effects of mitochondrial transplantation after IRI, but its clinical translation remains limited. Further investigations are thus required to explore the mechanisms of action and patient outcomes in critical settings after mitochondrial transplantation.

*Systematic review registration* The study was registered at UMIN under the registration number UMIN000043347.

**Supplementary Information:**

The online version contains supplementary material available at 10.1186/s12967-021-02878-3.

## Background

Mitochondrial transplantation is a therapeutic approach that involves injection of healthy mitochondria into damaged organs. Recent evidence has shown that the physiological properties of healthy mitochondria provide the possibility of replacing damaged mitochondria [1, 2], suggesting that replacement of damaged mitochondria with healthy mitochondria may protect cells against further injury [3]. Furthermore, mitochondria might also be actively released into the extracellular space and potentially be transferred from cell to cell in the central nervous system [4]. This heightened interest in mitochondrial therapy calls for a deeper understanding of the mechanisms underlying mitochondrial transfer, uptake, and cellular protection [5].

Alterations in mitochondrial properties and functions play a role in modulating the development of ischemia–reperfusion injury (IRI) in a variety of critical illnesses such as sudden cardiac arrest, stroke, myocardial infarction, and major organ transplants [6–12]. IRI alters the mitochondrial electron transport chain, produces an excess of reactive oxygen species, affects mitochondrial calcium transport and calcium concentration, drives apoptotic pathways, changes mitochondrial fission/fusion dynamics and their shape, and activates mitophagy pathways [13–21]. These pathophysiological alterations lead to neurocognitive sequelae, multiple organ failure, skeletal muscle dysfunction, dysregulation of immune cell responses, and release of mitochondrial DNA, which is now recognized as a trigger for systemic inflammatory responses and a prognostic marker in critical illness [20]. Therefore, the development of “mitochondria-targeting” approaches is an unmet medical need to improve the prognosis of critically ill patients.

Since the first study demonstrating mitochondrial transplantation as a promising therapy in 2009 [3], the effects of mitochondrial transplantation have been reported in different animal models of critical illness [4, 22–25]. However, the current evidence for the beneficial effects of mitochondrial transplantation in both preclinical and clinical settings has not been summarized systematically. Considering that systematic reviews (SR) of animal studies have been recently highlighted for improving the transparency and accessibility of the available evidence [26, 27], SRs of both preclinical and clinical efficacy studies are extremely valuable for promoting clinical translation [26]. Therefore, we conducted a systematic review of the relevant animal and human studies to summarize the current evidence of mitochondrial transplantation as a novel therapeutic strategy for IRI under critical conditions.

## Methods

This study complied with the recommendations for conducting and reporting systematic reviews as set forth by the Preferred Reporting Items for Systematic Reviews and Meta-Analyses (PRISMA) statement [28]. We developed a protocol before conducting the analysis and registered it in the UMIN-CTR (registration no. UMIN000043347). The PRISMA checklist is shown in Additional file 1.

### Search strategy

A comprehensive search of three major databases of biomedical publications was performed on February 17, 2021 as follows: MEDLINE (source: PubMed, 1966 to February 2021), Cochrane Central Register of Controlled Trials (through February 2021), and Embase (1974 to February 2021). We developed a search strategy using a combination of the following keywords and Medical Subject Heading (MeSH)/Emtree terms: “((("Mitochondria"[MeSH Terms]) OR ("Mitochondrial"[tiab])) AND ("transplantation"[MeSH Terms] OR "Mitochondrial transplantation"[tiab])) AND (("Craniocerebral Trauma"[MeSH Terms]) OR (("Reperfusion Injury"[MeSH Terms]) OR "Ischemia"[MeSH Terms] OR "Ischemi*"[tiab] OR "Ischemia–reperfusion"[tiab] OR "Heart arrest"[MeSH Terms])).” We did not apply any language restrictions to the electronic search. Citations were stored, and duplicates were removed using EndNote software (Thomson Reuters, Toronto, Ontario, Canada) and Rayyan software [29].

### Study selection and inclusion criteria

Two independent reviewers (KH and RT) screened the abstracts and titles of the studies, and subsequently reviewed the full-text articles for inclusion using Rayyan software [29].

We included studies with the following characteristics:

1. Study type: investigations in both laboratory and clinical settings
2. Target models/diseases: critical illness related to IRI, including myocardial infarction, organ transplantation, cardiac arrest, and acute brain injury. Studies on chronic central nervous system diseases, and on ex vivo animal models were excluded.
3. Intervention: intraarterial or intravenous injection, of isolated mitochondria, or their direct injection into the region at risk. Studies that used cell-mediated transfer of mitochondria as a transfer method and demonstrated transfer under cell culture conditions were excluded.
4. Control: Placebo or no intervention (without mitochondrial transplantation).
5. Outcome: biological or physiological measures of the IRI.

### Data extraction

Two independent reviewers (KH and RT) extracted the data using a standardized data extraction form, with disagreements resolved by discussion and consensus. To ensure that no duplicate data were analyzed, the reviewers assessed the studies carefully when multiple publications by the same investigator were found. We identified the following information for each study: primary author’s name, publication year, experimental model or patient population, randomization, blinded assessment, types of mitochondrial transplantation, and the main outcome. As the type and range of studies varied widely and were mostly animal models, we did not undertake formal quality assessment using an existing tool. The risk of bias across studies was not assessed. Instead, we qualitatively documented the potentially important limitations for each study. Evidence synthesis was descriptive because the data were not suitable for meta-analysis.

## Results

### Literature search

**Fig. 1:**
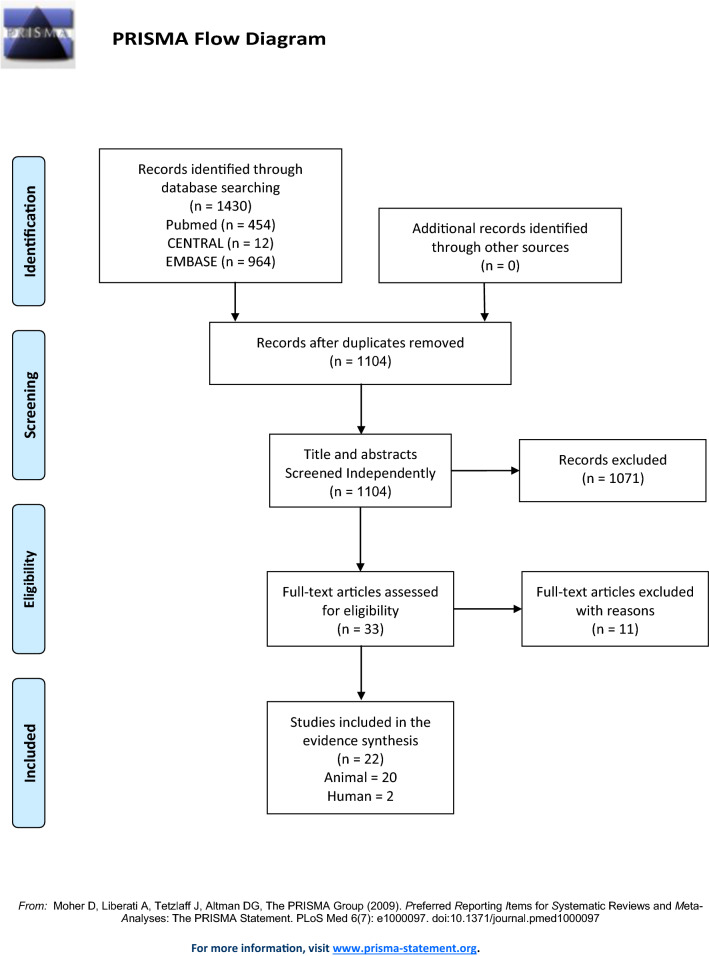
PRISMA flow diagram

Figure 1 shows the PRISMA flowchart of study selection in the systematic review. The search identified 1430 records. After removing 326 duplicate articles, 1104 articles were selected for title and abstract screening. Following screening, 33 articles were selected for full-text analysis. After full-text review, 11 articles did not meet the inclusion criteria and 22 articles were included in the review; of these, 20 were animal studies and 2 were human studies.

### Animal studies

A summary of the animal studies eligible for this review is presented in Table 1.

**Table 1:** Summary of the animal studies eligible for this review

Species	Type of model	Target Organ	Source of mitochondria	Transplantation method, dose, and timing	Randomization	Blinded assessment	Main outcomes	Refs
C57BL6 mice (male)	Focal ischemia (MCAO)	Brain	Allograft: Placenta	IV, 100 μg, Immediately after Reperfusion	Yes	Yes	Decreased infarct size 72 h post-ischemia	[25]
Wistar rat	Focal ischemia (MCAO)	Brain	Xenograft: hUC-MSCs	Direct ICVs, 10 μL of healthy mitochondria isolated from 3 × 10^7^ MSCs, After reperfusion (within 10 min)	Not reported	Not reported	Reduced Infarct size 72 h after ischemia; Improved motor function after 24 h	[31]
SD rat (male)	Focal ischemia (MCAO)	Brain	Autograft: Pectoralis major muscle	Direct ICVs, 5 × 10^6^, Immediately after reperfusion	Yes	Yes	Improved motor functions after MCAO, with reduced infarct volume and apoptosis	[32]
SD rats (male)	Focal ischemia (MCAO)	Brain	Xenograft: BHK-21 cells	Direct IC, 75 μg or IA (femoral), 750 μg, 24 h post-MCAO	Not reported	Not reported	IC and IA reduced infarct size 4 weeks post-ischemia and improved functional rotarod and grip strength performance for up to 1-month post-transplantation	[33]
1) bEnd3 and PC12 cell 2) C57BL6 mice (male)	1) OGD, 2) TBI	1) Cell, 2) Brain	Allograft: BDMts	1) Co-culture 2) IC into the ipsilateral cortex, 1.1 × 10^7^ mitochondria/μL × 10 μL, 10 min post-TBI	Yes	Yes	1) In vitro: improved cellular respiration and synaptic plasticity 2) In vivo: Reduced apoptosis, BBB damage, and brain edema	[30]
Yorkshire pigs (female)	Focal ischemia	Heart	Autograft: Pectoralis major muscle	IA (coronary), 1 × 10^9^, 120 min after reperfusion	Yes	Not reported	Reduced myocardial infarct size and enhanced regional and global myocardial function post-reperfusion	[34]
Yorkshire Pigs (female)	Focal ischemia	Heart	Autograft: Pectoralis major muscle	Subendocardial injection 8 times, 1.3 × 10^7^ mitochondria per injection site, 1 min before reperfusion	Yes	Not reported	No change in inflammatory and cytokine activation markers; decreased infarct size but no change in global function	[36]
Yorkshire swine (female)	Focal ischemia	Heart	Autograft: Pectoralis major muscle	IA (coronary), 1 × 10^9^, Immediately on reperfusion	Not reported	No	Improved myocardial function, perfusion, and infarct size	[37]
Yorkshire Pigs (female)	Focal ischemia	Heart	Autograft: Pectoralis major muscle	Single IA (coronary): 1 × 10^9^, 15 min before regional ischemia Serial IA (coronary): 1 × 10^9^ mitochondria × 10 injections, Every 5 min since 60 min before ischemia	Yes	Yes	Reduced myocardial infarct size, improved myocardial function; no difference between single and serial injections	[38]
New Zealand White rabbits (female)	1) Image study: Global or regional ischemia 2) Function study; regional ischemia	Heart	1) Xenograft: Human cardiac fibroblasts 2) Autograft: Liver	2) IA (coronary), 1 × 10^8^, Upon reperfusion	Not reported	Yes	1) Mitochondria were observed in interstitial spaces, associated with blood vessels, and cardiomyocytes 2) Reduced infarct size and enhanced myocardial function	[39]
New Zealand white rabbits (male)	Focal ischemia	Heart	Autograft: Pectoralis major muscle	Direct injection 8 times, 1.2 × 10^6^ per injection site, 1 min before reperfusion	Not reported	Yes	Reduced myocardial infarct size and enhanced regional myocardial function post-reperfusion	[40]
C57BL/6 J mice (male)	Focal ischemia	Heart	Allograft: Gastrocnemius muscle	IA (coronary), 1 × 10^8^,10 min before organ harvest and 5 min after transplantation	Not reported	Yes	Enhanced graft function and decreased graft tissue injury	[41]
C57BL/6 mice (male)	Focal ischemia	Heart	Not reported	Direct injection at myocardium of the left ventricle, 5 × 10^4^, During 24 h perfusion at 4 different points	Not reported	Not reported	Mitochondrial transplantation inhibited cardiomyocyte apoptosis in vitro In vivo transplantation of Alda-1-treated mitochondria limited infarction size after I/R injury	[42]
Yorkshire pigs (female)	Global ischemia	Heart	1) 1^st^, Autograft: Pectoralis major muscle 2) 2^nd^, Allograft: swine cardiac fibroblast cell	IA (coronary), 5 × 10^9^, 1) 15 min post-reperfusion 2) 2 h post-reperfusion	Yes	Yes	Preserved myocardial function and oxygen consumption and, decreased infarct size	[35]
Wistar rats (male)	Focal ischemia	Kidney	Autograft: Pectoralis major muscle	IA (renal), 7.5 × 10^6^, 5 min before reperfusion	Not reported	Not reported	Increased renal function, renal cell repair, and proliferation capacity	[43]
Yorkshire pigs (female)	Focal ischemia	Kidney	Autograft: Sternocleidomastoid muscle	Single IA (renal artery), 1 × 10^9^, Immediately at reperfusion	Yes	Yes	No safety issues detected Increased GFR and urine output, decreased serum creatinine and BUN	[44]
C57BL/6 J mice (male)	Focal ischemia	Hindlimb	Allograft: Muscle	Direct injection, 1 × 10^6^–1 × 10^9^ per gram muscle wet weight, 15 min after reperfusion	Not reported	Yes	Decreased infarct size and apoptosis; improved hindlimb function	[45]
C57BL/6 J mice (male)	Focal ischemia	Lung	Allograft: Gastrocnemius muscle	IA (pulmonary), 1 × 10^8^, or Aerosol delivery to whole lung by nebulization, 3 × 10^8^, Immediately at reperfusion	Yes	Yes	Both delivery methods improved lung mechanics and decreased lung tissue injury	[46]
SD rats (male)	Focal ischemia	Spinal cord	Allograft: Soleus muscle	IV (jugular), 100 μg, 5 min before reperfusion	Yes	Yes	Attenuated inflammatory, ER stress, and neuro-apoptotic reactions Improvement in motor function	[47]
SD rats (male)	Focal ischemia	Liver	Allograft: Liver	Portal vein, 10 mg, Upon reperfusion	Yes	Not reported	^31^P-MRS showed that the hepatic levels of ATP and NADH were higher in the m-Mito group than in the IRI group. The m-Mito group decreased the liver injury score and inflammatory cell infiltration in liver compared to the IRI group	[48]

SD, Sprague–Dawley; hUC-MSCs, human umbilical cord derived mesenchymal stem cells; MCAO, middle cerebral artery occusion; OGD, glucose oxygen deprivation; TBI, traumatic brain injury; BHK-21, Baby hamster kidney fibroblast; BDMt, brain-derived mitochondria; ICV, intracerebroventricular injection; IC, intracerebral injection; IA, intra-arterial injection; BBB, blood brain barrier; GFR, glomerular filtration rate; BUN, blood urea nitrogen; ER, endoplasmic reticulum; IRI, ischemia reperfusion injury; ^31^P-MRS, ^31^P-magnetic resonance spectroscopy; m-Mito, melatonin pretreated-mitochondrial transplantation

### Brain IRI model

Five experimental models targeting brain injury were generated in mice [25] and rats [30–33], including four focal brain ischemia models (middle cerebral artery occlusion [MCAO]) [25, 31–33] and one traumatic brain injury (TBI) model [30]. The types of transplantation used were autologous [32], allograft [25, 30], and xenograft [31, 33]. The types of administration were direct or near-direct injection [30–33] and intravenous [25].

Four animal studies demonstrated the benefits of mitochondrial transplantation in the setting of stroke. Using a 60 min focal carotid occlusion model in C57BL6 male mice, Nakamura et al. [25] assessed the impact of transfusing mitochondrial-enriched fractions obtained from snap-frozen placenta on the infarct size. Treatment with placental mitochondria significantly decreased the infarct size assessed using 2,3,5-triphenyltetrazolium chloride (TTC) staining at 72 h after reperfusion; further, fluorescent Mitotracker Deep Red imaging demonstrated diffuse mitochondrial uptake in the brain, lung liver, and kidney tissues at 2 h after treatment [25]. Excess reactive oxygen species and oxidative stress are major contributors to brain damage following acute ischemic stroke. Pourmohammadi-Bejarpasi et al. [31] reported the intracerebroventricular transplantation of isolated mitochondria from human umbilical cord-derived mesenchymal stem cells (hUC-MSCs) in a rat model of MCAO. MCAO occlusion in rats for 70 min followed by reperfusion and mitochondrial transplantation demonstrated normal brain cytoarchitecture and neurons with a decreased number of pyknotic cells [31]. Brain injury after acute stroke can result in astrocyte hypertrophy, and the subsequent release of glial fibrillary acidic protein (GFAP) can lead to scar formation, thus limiting neuronal recovery. Zhang et al. [32] demonstrated the neuroprotective effects of autologous mitochondrial transplantation obtained from the pectoralis major to the lateral ventricles after 90 min of MCAO. Twenty-four hours after MCAO, the number of viable mitochondria observed in the cerebrospinal fluid were increased, which resulted in improved neurological outcomes, suggesting the potential application of mitochondrial transplantation after stroke. Infusion of isolated mitochondria in the lateral ventricles post-stroke resulted in increased mitochondrial accumulation in the penumbra with an increase in overall ATP content and in the expression of complex IV. Post-MCAO transplantation of mitochondria also reduced apoptosis, attenuated astrogliosis with increased neurogenesis, and decreased the brain infarct volume [32]. Huang et al. [33] used a male rat MCAO cerebral stroke model to compare the effects of direct intracerebral injection *vs*. intra-femoral transfusion on functional status and infarct size. Intracerebral and intra-femoral transplantation improved the functional rotarod and grip strength performance measured up to 1-month post-transplantation, significantly decreased the lesion size as estimated using Terminal deoxynucleotidyl transferase dUTP nick end labeling (TUNEL) assay, and showed diffuse distribution of the grafted mitochondria in neurons, astrocytes, and microglia. Mitochondria treated with antimycin A and oligomycin failed to provide equivalent protection against oxygen glucose deprivation (OGD)-induced stress, suggesting that transplantation benefits require intact mitochondrial function [33].

One animal study demonstrated the benefits of mitochondrial transplantation in the setting of TBI. TBI is known to result in inflammatory and oxidative stress as well as mitochondrial dysfunction that occurs shortly after the initial impact. Using both a cellular glucose-oxygen deprivation model in bEnd3 and PC12 cells and an in vivo mouse model of TBI using a controlled cortical impact device, Zhang et al. [30] suggested that administration of exogenous brain-derived mitochondria improved cellular respiration and the expression of proteins associated with synaptic plasticity in vitro, along with increased angiogenesis; reduction in apoptosis, brain edema, and blood brain barrier leakage; and improved long-term potentiation in the hippocampus, at 7 days after in vivo TBI.

All the studies showed improvement in the primary outcome with mitochondrial transplantation. All the above-mentioned studies demonstrated an improvement in brain function following mitochondrial transplantation. Of these, two out of five studies did not report whether the study was randomized or if the study was conducted in a blinded manner; the remaining studies included information regarding both randomization and blinding. The sources of donor organs, types of transplantation, timing of transplantation, and transplant methods varied across all studies.

### Cardiac IRI model

Nine experimental models of cardiac IRI were undertaken in pigs [34–38], rabbits [39, 40], and mice [41, 42], including seven focal ischemia models [34, 36–42] and one global ischemia model [35]. The types of transplantation were autologous [34–40] and allograft [41]. Administration was intraarterial [34, 35, 37–39, 41] and through direct injection [36, 40].

We reviewed seven animal studies that demonstrated the benefits of mitochondrial transplantation in focal cardiac ischemia models. Blitzer et al. [34] used Yorkshire pigs that underwent 30 min of ischemia, which was induced by snaring the left anterior descending artery, to test the efficacy of delayed mitochondrial transplantation. After the initial 120 min of reperfusion, swine were randomly assigned to receive either autologous mitochondria (1 × 10^9^ in 5 mL of vehicle) or vehicle only (5 mL) delivered antegrade as a bolus to the left coronary ostium. Echocardiographic analysis demonstrated that the hearts of pigs receiving delayed mitochondrial transplantation after IRI showed enhanced ejection fraction, fractional shortening, and fractional area change at 240 min of reperfusion compared with those in the untreated pigs. Although no significant differences were found between the groups in the area at risk, the infarct size was significantly decreased in the hearts of treated pigs [34]. Kaza et al. [36] injected autologous mitochondria into the area at risk (AAR) of the heart after 24 min of temporary regional ischemia, which was induced by snaring the circumflex artery in a pig model. The mitochondria isolated from the dissected pectoralis major muscle were injected into the AAR (1.3 × 10^7^ per injection site × 8 injection site), which significantly enhanced myocardial cell viability and reduced the infarct size. Magnetic resonance imaging showed that the injected mitochondria were present for at least four weeks after the injection [36]. Shin et al. [37] demonstrated that autologous mitochondria (1 × 10^9^ mitochondria) injected into the left coronal artery after 30 min of regional ischemia, which was induced by temporary snaring of the mid left anterior descending artery, significantly improved myocardial function, coronary blood flow, and infarct size. The authors thus concluded that intracoronary delivery of mitochondria is a safe and efficacious therapy for myocardial ischemia–reperfusion injury [37]. Guariento et al. [38] performed autologous mitochondrial transplantation as a therapeutic strategy for prophylactic myocardial protection in a porcine model of regional IRI. Yorkshire pigs received vehicle or mitochondria either as a single bolus (1 × 10^9^: MT_S_) or serially (10 injections of 1 × 10^9^ at 5 min intervals over 60 min: MT_SS_) prior to cardiac IRI. MT_S_ and MT_SS_ significantly enhanced coronary blood flow and ejection fraction and significantly reduced the infarct size. However, no significant difference in AAR was observed between the treatment groups. The authors suggest that preischemic mitochondrial transplantation, either by single or serial administration, facilitates myocardial protection from IRI by significantly reducing the infarct size and enhancing global and regional function [38]. Cowan et al. [39] used New Zealand White rabbits to investigate whether exogenous mitochondria can be effectively delivered through the coronary vasculature to protect the ischemic myocardium, and studied the fate of these transplanted organelles in the heart. Xenografted mitochondria were observed in the interstitial spaces and were associated with the blood vessels and cardiomyocytes. They also found that autologous liver-derived mitochondria markedly reduced the infarct size and enhanced myocardial function [39]. Masuzawa et al. [40] transplanted mitochondria (9.7 ± 1.7 × 10^6^/mL), which were autologously derived from the pectoralis major muscle, in rabbits subjected to regional ischemia, 1 min prior to reperfusion. Regional ischemia was achieved by temporarily narrowing the left anterior descending artery for 30 min. The animals were then allowed to recover for 4 weeks to measure their heart function. Mitochondrial transplantation significantly decreased the infarct size, creatine kinase MB levels, cardiac troponin-I levels, and apoptosis in the RI zone compared with the vehicle treatment group. The authors also showed that in vivo and in vitro transplanted mitochondria were observed in the interstitial spaces and were internalized by cardiomyocytes at 2–8 h after transplantation. The transplanted mitochondria enhanced oxygen consumption, high-energy phosphate synthesis, and the induction of cytokine mediators and proteomic pathways that are important in preserving myocardial energetics, cell viability, and enhanced post-infarct cardiac function [40]. In a murine heart transplantation model, Moskowitzova et al. [41] reported that 1 × 10^8^ allogeneic mitochondria isolated from the gastrocnemius muscle were delivered antegrade to the coronary arteries via injection to the coronary ostium before harvesting the donor heart and after transplantation. Mitochondrial therapy (1 × 10^8^ in respiratory buffer) prolonged the cold ischemia time, significantly enhanced graft function, and decreased graft tissue injury [41].

We found one study that investigated the potential therapeutic effects of pretreating donor mitochondria for mitochondrial transplantation in myocardial IRI. Aldehyde dehydrogenase 2 (ALDH2) plays a key role in regulating mitochondrial homeostasis. Sun et al. [42] investigated the potential effects of Alda-1, an ALDH2 activator, on mitochondrial transplantation in myocardial IRI. In vitro, mitochondrial transplantation inhibited the cardiomyocyte apoptosis induced by hypoxia-reoxygenation exposure, independent of Alda-1 treatment. In vivo transplantation of Alda-1-treated mitochondria limited the IRI as assessed by echocardiography, 2,3,5-triphenyltetrazolium chloride (TTC) staining, and imaging studies.

One animal study demonstrated the benefits of mitochondrial transplantation in a global cardiac ischemia reperfusion model. In a study by Guariento et al. [35] in 2020, Yorkshire pigs received vehicle or autologous mitochondria as a single bolus (5 × 10^9^ MT), after 15 min of reperfusion. Another group of animals (serial injection of mitochondria [MT_S_]) received a second injection of mitochondria (5 × 10^9^) after 2 h of ex situ heart perfusion, and was reperfused for an additional 2 h. Both the MT and MT_S_ groups showed significantly increased left ventricle function (ventricular peak developed pressure, maximal ventricular pressure rise, fractional shortening), increased myocardial oxygen consumption, and decreased infarct size at 4 h post-reperfusion compared with the vehicle-treated group [35].

We did not find any studies that failed to demonstrate an improvement in cardiac function after mitochondrial transplantation. Seven of nine studies did not report either randomization or blinded assessment. In most studies, mitochondria that were autologously derived from the pectoralis major muscle were injected via the coronary artery.

### Kidney IRI model

There were two studies on experimental models of kidney focal ischemia in rats [43] and pigs [44], in which autologous mitochondria were injected intra-arterially.

Jabbari et al. [43] demonstrated that mitochondrial autografts from healthy muscle cells to injured kidney cells through injection into the renal artery prevented renal tubular cell death, restored renal function, ameliorated kidney damage, improved the regenerative potential of renal tubules, and decreased IRI-induced apoptosis [43]. Doulamis et al. [44] investigated the safety and efficacy of autologous mitochondrial transplantation by intra-arterial injection for renal protection in a swine model of bilateral renal IRI. They found that the injected mitochondria were rapidly taken up by the kidneys. After 24 h of reperfusion, the estimated glomerular filtration rate and urine output were significantly increased, whereas the serum creatinine and blood urea nitrogen levels were significantly decreased by mitochondrial transplantation, along with improved acute histopathological changes. The authors thus suggested that their results provide a basis for clinical translation and will enhance the armamentarium of clinicians for the treatment of acute kidney injury [44].

### Other IRI models

Studies on mitochondrial transplantation have also been conducted in other experimental models including acute limb ischemia [45], lung IRI [46], spinal cord injury [47], and acute liver injury [48] models. Orfany et al. [45] used a C57BL/6 J male mouse acute limb ischemia model to compare the impact of dose-dependent mitochondrial injection (1 × 10^6^, 1 × 10^7^, 1 × 10^8^, and 1 × 10^9^ mitochondria/g muscle) after IRI with f-Rhodamine 6G-labeled mitochondria. Positron emission tomography imaging demonstrated diffuse mitochondrial uptake into the injected muscle group and muscle histology showed significantly improved infarct size; further, dose-dependent significant differences were achieved between the lowest and highest dosing regimens. Mitochondrial injections were associated with improved functional status by visual gait scoring and increased IL-10 expression by multiplex analysis [45]. Moskowitzova et al. [46] investigated the efficacy of mitochondrial transplantation in a murine lung IRI model. Male C57BL/6 J mice received either vehicle or vehicle-containing mitochondria either by vascular delivery through the pulmonary artery or by aerosol delivery via the trachea (nebulization). At 24 h after reperfusion, lung function was found to be increased, whereas tissue injuries were significantly decreased in the mitochondria-treated groups compared with the respective vehicle groups. No significant differences in cytokine and chemokine levels were observed between the groups. The authors thus concluded that mitochondrial transplantation by vascular delivery or nebulization improved lung mechanics and decreased lung tissue injury [46]. Fang et al. [47] tested the neuronal protective effect of transplanting viable mitochondria into the ischemic spinal cord in rats. Mitochondria (100 µg) were isolated from the soleus muscle and delivered through the jugular vein before reperfusion. They found that mitochondrial transplantation significantly reduced neuronal apoptosis and improved locomotor function in rats with spinal cord ischemia by suppressing oxidative stress and endoplasmic reticulum (ER) stress [47]. Ko et al. [48] investigated whether ^31^P-magnetic resonance spectroscopy (^31^P-MRS) could accurately identify the protective effects of transplanting melatonin-pretreated mitochondria (m-Mito) on acute liver IRI in rats. At 72 h after acute liver IRI, ^31^P-MRS showed that the hepatic levels of ATP and NADH were significantly higher in the m-Mito group than in the IRI group. Histopathology revealed that the liver injury score and inflammatory cell infiltration in the liver parenchyma were significantly decreased with the m-Mito-transplanted IRI group [48].

All studies demonstrated improvement in IRI following mitochondrial transplantation. In most studies, mitochondria derived from skeletal muscles were used for allograft transplantation. The transplantation method and timing differed depending on the experimental model used.

### Human studies

In this systematic review, we did not find any randomized controlled trials of mitochondrial transplantation in humans. We identified two human studies that used mitochondrial transplantation, and these were reported by the same study group. The group first published a single-arm interventional case series with autologous mitochondrial transplantation in five pediatric patients who required central extracorporeal membrane oxygenation (ECMO) support, and showed that mitochondrial transplantation is feasible and safe [49]. Thereafter, in 2020, the same group reported their further experience with injection of autologous mitochondria into the myocardium of pediatric patients in a single-center retrospective study [50].

At Boston Children’s Hospital (BCH) from May 2002 to December 2018, 10 pediatric patients with severe heart disease who required central ECMO support for IRI-associated myocardial dysfunction after cardiac surgery were eligible for autologous mitochondrial transplantation. These 10 subjects were compared with 14 historical controls.

Mitochondria were harvested from non-ischemic rectus abdominis muscle, and the isolation was performed within 20–30 min under sterile conditions in the cardiac intensive care unit or operating room. A 6 mm × 6 mm piece of heathy rectus abdominis muscle was harvested and mitochondria were isolated, yielding approximately 2 × 10^10^ viable and respiration competent mitochondria from 0.18 ± 0.04 g (wet weight) of tissue sample. The isolated mitochondria were suspended in 1 mL of respiration buffer at a concentration of approximately 1 × 10^8^ to 1 × 10^9^ particles/mL. During the same intervention, mitochondria were delivered by direct injection using a tuberculin syringe (28-gauge needle) to the myocardium affected by IRI, as identified by epicardial echocardiography. After the procedure, cardiac function assessed by speckle tracking echocardiography was reviewed by two blinded investigators for median circumferential strain and qualitative assessment.

No patients had adverse short-term complications related to mitochondrial injection (arrhythmia, intramyocardial hematoma, or scarring). Blood parameters (white blood count, serum lactate, creatinine, and blood urea nitrogen) did not differ between patients who underwent mitochondrial transplantation and those who did not receive mitochondrial transplantation. Patients subjected to mitochondrial transplantation were more likely to successfully wean from ECMO and exhibited better ventricular strain magnitude at the time of initial decannulation (23.0% *vs*. 16.8%, P = 0.03) and lower overall occurrence of cardiovascular events (20% *vs*. 79%, P < 0.01) compared to the control group. Kaplan–Meier curves and Cox regression analysis models showed a higher composite estimated risk of cardiovascular events in the control group (hazard ratio, 4.6; 95% confidence interval, 1.0–20.9; P < 0.04) [50].

## Discussion

### Summary of main results

We identified 20 animal and 2 human studies to provide current evidence for mitochondrial transplantation as a treatment for IRI. In the animal studies, 14 of 20 (70.0%) studies focused on IRI models of either the brain or heart, and 12 (60.0%) studies were performed in a blinded manner. Both autograft and allograft transplantation were used in 17 (85.0%) animal studies; the study designs were heterogeneous in terms of route of administration, timing of transplantation, and the dosage used. All animal studies reported that mitochondrial transplantation markedly mitigated IRI in the targeted tissues, but there was variation in the biological biomarkers and pathological changes. Of these, two studies demonstrated the beneficial effects of pretreating mitochondria as donors on IRI. Overall, irrespective of the experimental model, none of the studies failed to demonstrate an improvement in functional outcomes after mitochondrial transplantation. Further studies are warranted to elucidate the best methods, the best donors, or the best timing of mitochondrial transplantation for IRI.

In this systematic review, we found only two studies reported by the same group, but did not find any randomized controlled trials regarding the effect of mitochondrial transplantation on IRI. The latest study reported was a single-center, non-randomized, retrospective study with historical controls, which should be noted as the major limitations of the study. Further randomized controlled trials are thus required to demonstrate the impact of mitochondrial transplantation in patients with IRI.

### Potential mechanisms underlying the beneficial effects of mitochondrial transplantation on IRI

**Fig. 2:**
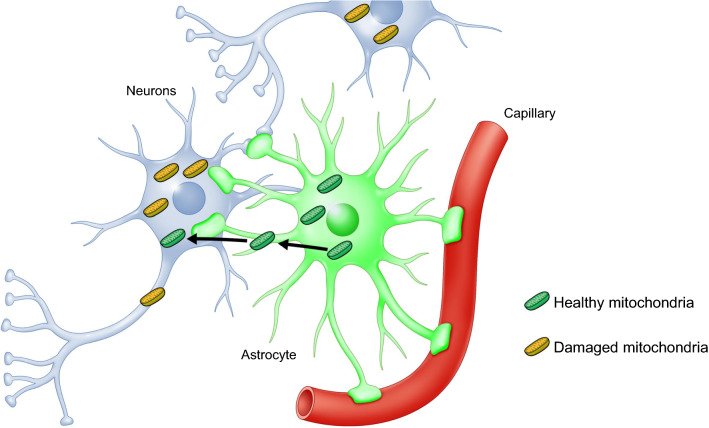
Mitochondrial transfer from astrocytes to damaged neurons following ischemic insult

The mechanisms responsible for mitochondrial transplantation in IRI are yet to be fully elucidated. Astrocytes play a wide variety of roles in the regulation of neurodevelopment, neurotransmission, blood brain barrier formation, and in protection against oxidative stress and excitotoxicity. Recent studies have shown that astrocytes can release and transfer extracellular mitochondrial particles that enter damaged neurons to support neuroprotection and neurorepair through calcium-dependent CD38 signaling [4, 51] (Fig. 2).

In animal models of brain ischemia, transplanted mitochondria were found to be incorporated into neurons [32], astrocytes [33], and microglial cells [33] of the peri-infarct area, resulting in increased overall ATP content and increased expression of complex IV [32]. Mitochondrial transplantation significantly attenuates cellular oxidative stress, apoptosis [32], and decreased astrogliosis [31, 32] and microglial activation [31], and promotes neurogenesis after ischemia [32]. Furthermore, fluorescent imaging revealed that in addition to the ischemic brain, infused labeled mitochondria were found in various organs including the lung, liver, kidney, and heart [25]. Thus, further studies are required to determine how intravenous infusion of mitochondria affects other organs outside the central nervous system after stroke. In models of cardiac IRI, transplanted mitochondria have been shown to act both extra- and intra-cellularly [22]. Injected mitochondria were found to be localized near the site of delivery, whereas vascular perfusion of mitochondria resulted in rapid and extensive dispersal throughout the heart [39]. Transplanted mitochondria increased the total tissue ATP content and ATP synthesis [40] as well as enhanced myocardial cell viability [36]. This increase in high-energy molecules acts to improve cardiac function rapidly. Further, the transplanted mitochondria enhanced the biological pathways that are important for preserving myocardial energetics and cell viability [40]. Proteomic analysis has shown upregulation of proteomic pathways for energy production, mitochondrial function, and cellular respiration [40]. Mitochondrial transplantation decreased pro-inflammatory markers [40, 47], increased anti-inflammatory cytokines [45], and inhibited endoplasmic reticulum stress and caspase-3 expression [47]. Mitochondrial transplantation did not alter the levels of pro-inflammatory cytokines [36, 45, 46]. Moreover, metabolomic analysis has indicated that mitochondrial electron transport chain was in the top 10 pathways involved in the altered metabolic profile of the hearts that received mitochondria [35].

**Fig. 3:**
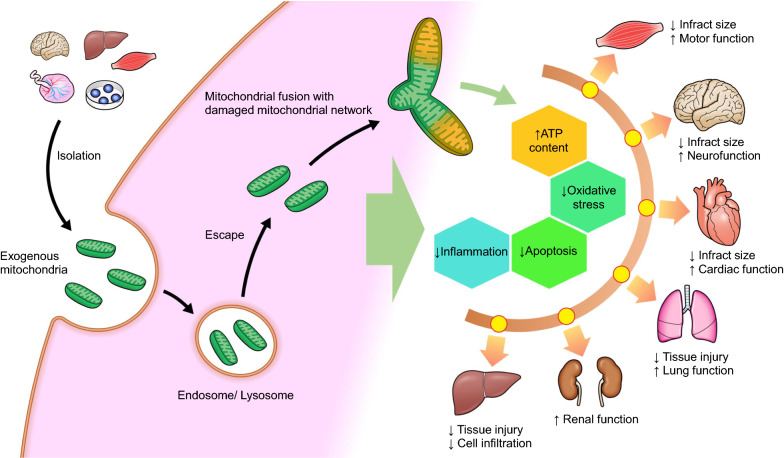
Potential mechanisms by which mitochondrial transplantation improves outcomes in ischemia–reperfusion injury under critical conditions

The potential mechanisms by which mitochondrial transplantation improves IRI outcomes are shown in Fig. 3. Although the precise mechanisms by which exogenous mitochondria can be taken up by cells remain undetermined, the transplanted exogenous mitochondria are found to successively progress through the endolysosomal system from early endosomes to late endosomes to lysosomes [52]. Most exogenous mitochondria escape from endosomal and lysosomal compartments and effectively fuse with endogenous cardiac cell mitochondria, along with an associated increase in ATP content, oxygen consumption rates, and replacement with depleted mitochondrial DNA [52, 53] (Fig. 3). However, further studies elucidating the molecular mechanism of exogenous mitochondria are warranted to guide the development of this innovative treatment.

From the viewpoint of clinical translation, a potential alternative source for viable, respiratory-competent mitochondria would be from a different individual (allogeneic) or different species (xenogeneic). Ramirez-Barbieri et al. investigated the immune and damage-associated molecular patterns (DAMPs)-associated response after injections of allogeneic mitochondria in a transplant rejection system of fully MHC-mismatched skin allografts. Their results demonstrated that there is no direct or indirect, acute or chronic alloreactivity, allorecognition or DAMPs reaction to single or serial injections of allogeneic mitochondria [54]. The same group has also shown that in a rabbit model of cardiac IRI the xenogeneic mitochondria transplantation upregulated only cytokines and chemokines which were associated with enhanced post-infarct cardiac function and were not related to alloresponse [40]. All other cytokines and chemokines showed identical levels to baseline at 1, 2, 3 and 4 weeks post mitochondrial injection [40].

### Strength of the review

A significant strength of this analysis is that, to the best of our knowledge, this study is the first systematic review to provide evidence regarding the potential beneficial effects of mitochondrial transplantation on IRI in critical diseases, in both preclinical and clinical settings. We performed a comprehensive search without any language restrictions. Furthermore, given that recent applications of SR to animal studies revealed poor quality (in terms of randomization and blinding) in most animal studies [26, 55–57], our SR of both preclinical and clinical studies enables scrutiny of the validity of preclinical evidence, helps prevent unnecessary duplication of animal studies, and promotes the clinical translation of this promising concept of mitochondrial transplantation.

### Limitations of the review

The current study has several limitations. First, as the concept of mitochondrial transplantation is in its early stages, we should acknowledge the significant possibility of publication bias, even in animal studies. We also cannot exclude reporting bias in both animal and human studies. This implies that the techniques used may be suboptimal. Second, we did not perform a quantitative meta-analysis because we considered that quantitative meta-analysis was not relevant for the small number of heterogeneous studies included; thus, our data synthesis is mostly descriptive. Third, although we tried to capture a wide range of studies, we may have missed potentially relevant literature. Finally, it should be noted that 12 out of 20 animal studies included in this review used rodent models. It is known that the rodents model has lesser similarities to humans than rabbits and pig models in terms of the physiological, hemodynamical, immune response, and metabolic responses [58, 59].

## Conclusion

In the present review, we have summarized the development and potential applications of mitochondrial transplantation in IRI. The studies included in our analysis have demonstrated the potential of mitochondrial transplantation in the context of IRI in different organs. The provided evidence supports the potential impact of mitochondrial transplantation on IRI in preclinical models, but the relevant clinical translational research remains extremely limited. Therefore, further investigations are required, especially in clinical settings, to explore the mechanism of action and the potential for clinical implementation of mitochondrial transplantation to improve patient outcomes.

## Supplementary Information

**Additional file 1**. PRISMA 2009 checklist RT.

## Data Availability

Not applicable.

## Abbreviations

IRI: Ischemia reperfusion injury
ECMO: Extracorporeal membrane oxygenation
SR: Systematic review
MeSH: Medical subject headings
MCAO: Middle cerebral artery occlusion
TBI: Traumatic brain injury
AAR: Area at risk
